# 1-(2,5-Dichloro-3-thien­yl)ethanone: infinite sheets mediated by O⋯Cl halogen bonds

**DOI:** 10.1107/S1600536810035154

**Published:** 2010-09-04

**Authors:** William T. A. Harrison, C. S. Chidan Kumar, H. S. Yathirajan, A. N. Mayekar, B. Narayana

**Affiliations:** a Department of Chemistry, University of Aberdeen, Meston Walk, Aberdeen AB24 3UE, Scotland; b Department of Studies in Chemistry, University of Mysore, Manasagangotri, Mysore 570 006, India; c SeQuent Scientific Limited, New Mangalore 575 011, India; d Department of Chemistry, Mangalore University, Mangalagangotri 574 199, India

## Abstract

In the title compound, C_6_H_4_Cl_2_OS, the acetyl group is almost coplanar with the thio­phene ring [dihedral angle = 4.01 (2)°]. In the crystal, short inter­molecular O⋯Cl contacts [2.9494 (14) and 3.1191 (14) Å] link the mol­ecules into infinite (100) sheets and aromatic π–π stacking [centroid–centroid separation = 3.5422 (10) Å] consolidates the packing.

## Related literature

For a related structure and background references, see: Jasinski *et al.* (2010 ▶). For a related structure, see: Wen & Rasmussen (2007 ▶). For reference structural data, see: Allen *et al.* (1987 ▶). For a discussion of halogen bonding, see: Metrangalo & Resnati (2001 ▶).
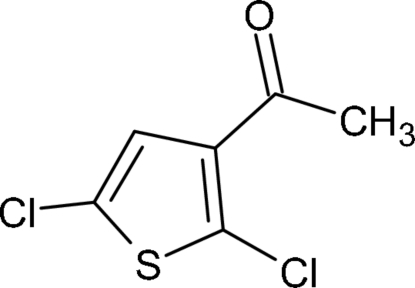


## Experimental

### 

#### Crystal data

C_6_H_4_Cl_2_OS
*M* *_r_* = 195.05
Orthorhombic, 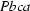

*a* = 13.0980 (3) Å
*b* = 7.1790 (1) Å
*c* = 16.3290 (3) Å
*V* = 1535.42 (5) Å^3^
*Z* = 8
Mo *K*α radiation
μ = 1.04 mm^−1^
*T* = 120 K
0.22 × 0.14 × 0.08 mm

#### Data collection

Nonius KappaCCD diffractometer
Absorption correction: multi-scan (*SADABS*; Bruker, 2003 ▶) *T*_min_ = 0.804, *T*_max_ = 0.922
12928 measured reflections
1758 independent reflections
1538 reflections with *I* > 2σ(*I*)
*R*_int_ = 0.050

#### Refinement

*R*[*F* ^2^ > 2σ(*F* ^2^)] = 0.031
*wR*(*F* ^2^) = 0.078
*S* = 1.07
1758 reflections
92 parameters
H-atom parameters constrained
Δρ_max_ = 0.34 e Å^−3^
Δρ_min_ = −0.28 e Å^−3^

### 

Data collection: *COLLECT* (Nonius, 1998 ▶); cell refinement: *SCALEPACK* (Otwinowski & Minor, 1997 ▶); data reduction: *DENZO* (Otwinowski & Minor 1997 ▶), *SCALEPACK* and *SORTAV* (Blessing, 1995 ▶); program(s) used to solve structure: *SHELXS97* (Sheldrick, 2008 ▶); program(s) used to refine structure: *SHELXL97* (Sheldrick, 2008 ▶); molecular graphics: *ORTEP-3* (Farrugia, 1997 ▶); software used to prepare material for publication: *SHELXL97*.

## Supplementary Material

Crystal structure: contains datablocks I, global. DOI: 10.1107/S1600536810035154/jj2050sup1.cif

Structure factors: contains datablocks I. DOI: 10.1107/S1600536810035154/jj2050Isup2.hkl

Additional supplementary materials:  crystallographic information; 3D view; checkCIF report

## Figures and Tables

**Fig. 1.**
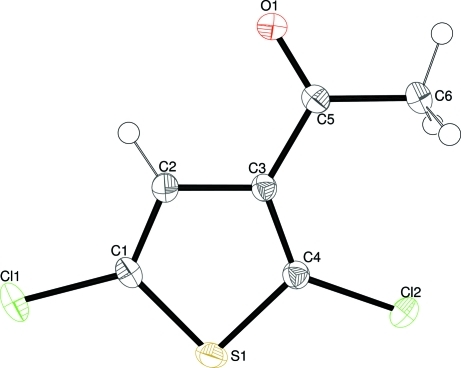
View of the molecular structure of (I) showing 50% displacement ellipsoids (arbitrary spheres for the H atoms).

**Fig. 2.**
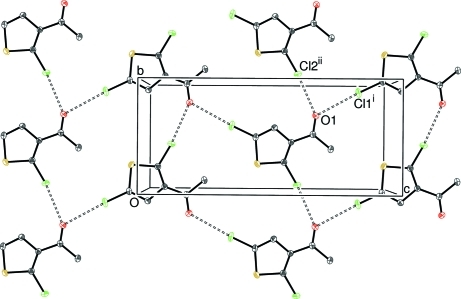
Partial packing diagram for (I) showing part of a (100) sheet arising from short O···Cl contacts (H atoms omitted for clarity). Symmetry codes: (i) x, 3/2–y, 1/2 + z; (ii) x, 1 + y, z.

## supplementary crystallographic information

### Comment

The structure of the title compound, (I), (Fig. 1), was determined as part of our ongoing studies (Jasinski *et al.*, 2010) of thiophene derivatives as possible candidates for non-linear optical materials.

The five-membered ring in (I) is almost planar (r.m.s. deviation = 0.002 Å) and the pendant atoms deviate from the ring plane by 0.019 (1)Å (Cl1), 0.011 (1)Å (Cl2), 0.110 (1)Å (O1), 0.026 (2)Å (C5) and -0.045(20Å (C6). The dihedral angle between C1/C2/C3/C4/S1 and C5/C6/O1 is 4.01 (2)°. Otherwise, the bond lengths for (I) fall within their expected ranges (Allen *et al.*, 1987) and are similar to those in related structures (Wen & Rasmussen, 2007).

In the crystal of (I), short O···Cl contacts of 2.9494 (14)Å and 3.1191 (14)Å are evident, compared to an expected van der Waals' separation of about 3.27Å for these atoms. If theses contacts are considered to be bonding interactions (Metrangalo & Resnati, 2001), then infinite (100) sheets result (Fig. 2). Seventeen-membered rings containining four O···Cl bonds are apparent within the sheet. The packing is consolidated by aromatic π-π stacking interactions, with a centroid–centroid separation of 3.5422 (10)Å between inversion-related thiophene rings.

### Experimental

2,5-Dichloro-3-acetylthiophene was obtained as a gift sample from SeQuent Scientific Ltd., New Mangalore, India. Colourless blocks of (I) were grown by the slow evaporation of a methanol solution (M.P.: 314–316 K).

### Refinement

The hydrogen atoms were geometrically placed (C—H = 0.95–0.98 Å) and refined as riding with *U*_iso_(H) = 1.2*U*_eq_(C) or 1.5*U*_eq_(methyl C). A rotating rigid-group model was applied to the methyl group.

### Crystal data

**Table:**

C_6_H_4_Cl_2_OS	*F*(000) = 784
*M**_r_* = 195.05	*D*_x_ = 1.688 Mg m^−^^3^
Orthorhombic, *P**b**c**a*	Mo *K*α radiation, λ = 0.71073 Å
Hall symbol: -P 2ac 2ab	Cell parameters from 12696 reflections
*a* = 13.0980 (3) Å	θ = 2.9–27.5°
*b* = 7.1790 (1) Å	µ = 1.04 mm^−^^1^
*c* = 16.3290 (3) Å	*T* = 120 K
*V* = 1535.42 (5) Å^3^	Cut block, colourless
*Z* = 8	0.22 × 0.14 × 0.08 mm

### Data collection

**Table:**

Nonius KappaCCD diffractometer	1758 independent reflections
Radiation source: fine-focus sealed tube	1538 reflections with *I* > 2σ(*I*)
graphite	*R*_int_ = 0.050
ω and φ scans	θ_max_ = 27.5°, θ_min_ = 2.9°
Absorption correction: multi-scan (*SADABS*; Bruker, 2003)	*h* = −17→17
*T*_min_ = 0.804, *T*_max_ = 0.922	*k* = −9→9
12928 measured reflections	*l* = −21→19

### Refinement

**Table:**

Refinement on *F*^2^	Primary atom site location: structure-invariant direct methods
Least-squares matrix: full	Secondary atom site location: difference Fourier map
*R*[*F*^2^ > 2σ(*F*^2^)] = 0.031	Hydrogen site location: inferred from neighbouring sites
*wR*(*F*^2^) = 0.078	H-atom parameters constrained
*S* = 1.07	*w* = 1/[σ^2^(*F*_o_^2^) + (0.0196*P*)^2^ + 1.1699*P*] where *P* = (*F*_o_^2^ + 2*F*_c_^2^)/3
1758 reflections	(Δ/σ)_max_ = 0.002
92 parameters	Δρ_max_ = 0.34 e Å^−^^3^
0 restraints	Δρ_min_ = −0.28 e Å^−^^3^

### Special details

**Table:**

Geometry. All e.s.d.'s (except the e.s.d. in the dihedral angle between two l.s. planes) are estimated using the full covariance matrix. The cell e.s.d.'s are taken into account individually in the estimation of e.s.d.'s in distances, angles and torsion angles; correlations between e.s.d.'s in cell parameters are only used when they are defined by crystal symmetry. An approximate (isotropic) treatment of cell e.s.d.'s is used for estimating e.s.d.'s involving l.s. planes.
Refinement. Refinement of *F*^2^ against ALL reflections. The weighted *R*-factor *wR* and goodness of fit *S* are based on *F*^2^, conventional *R*-factors *R* are based on *F*, with *F* set to zero for negative *F*^2^. The threshold expression of *F*^2^ > σ(*F*^2^) is used only for calculating *R*-factors(gt) *etc*. and is not relevant to the choice of reflections for refinement. *R*-factors based on *F*^2^ are statistically about twice as large as those based on *F*, and *R*- factors based on ALL data will be even larger.

### Fractional atomic coordinates and isotropic or equivalent isotropic displacement parameters (Å^2^)

**Table:**

	*x*	*y*	*z*	*U*_iso_*/*U*_eq_	
C1	0.36993 (13)	0.4974 (3)	0.44792 (10)	0.0181 (4)	
C2	0.36763 (13)	0.5824 (2)	0.52170 (11)	0.0178 (4)	
H2	0.3618	0.7134	0.5285	0.021*	
C3	0.37494 (12)	0.4537 (2)	0.58881 (10)	0.0153 (4)	
C4	0.38165 (13)	0.2736 (2)	0.56061 (11)	0.0172 (4)	
C5	0.37563 (13)	0.5245 (3)	0.67461 (11)	0.0181 (4)	
C6	0.37733 (14)	0.3927 (3)	0.74569 (11)	0.0227 (4)	
H6A	0.3730	0.4634	0.7969	0.034*	
H6B	0.4410	0.3210	0.7448	0.034*	
H6C	0.3191	0.3074	0.7418	0.034*	
O1	0.37515 (11)	0.69263 (19)	0.68546 (8)	0.0299 (3)	
S1	0.38011 (3)	0.25777 (6)	0.45563 (3)	0.02062 (14)	
Cl1	0.36483 (4)	0.60020 (7)	0.35316 (3)	0.02524 (14)	
Cl2	0.39243 (4)	0.06821 (6)	0.61358 (3)	0.02400 (14)	

### Atomic displacement parameters (Å^2^)

**Table:**

	*U*^11^	*U*^22^	*U*^33^	*U*^12^	*U*^13^	*U*^23^
C1	0.0175 (9)	0.0234 (10)	0.0134 (9)	−0.0004 (7)	−0.0011 (6)	0.0017 (7)
C2	0.0191 (9)	0.0176 (8)	0.0168 (9)	0.0001 (7)	0.0000 (7)	0.0017 (7)
C3	0.0154 (8)	0.0162 (8)	0.0144 (9)	0.0004 (6)	0.0010 (6)	0.0010 (7)
C4	0.0174 (9)	0.0179 (8)	0.0163 (8)	−0.0007 (7)	0.0008 (7)	0.0006 (7)
C5	0.0190 (9)	0.0198 (8)	0.0154 (9)	0.0003 (7)	0.0005 (6)	−0.0007 (7)
C6	0.0310 (11)	0.0227 (10)	0.0145 (10)	0.0001 (7)	0.0021 (7)	0.0022 (7)
O1	0.0548 (10)	0.0174 (7)	0.0175 (7)	0.0006 (6)	−0.0007 (6)	−0.0024 (6)
S1	0.0244 (3)	0.0215 (3)	0.0160 (3)	−0.00193 (17)	0.00004 (17)	−0.00486 (17)
Cl1	0.0251 (3)	0.0372 (3)	0.0134 (2)	0.00171 (19)	−0.00100 (16)	0.00583 (18)
Cl2	0.0332 (3)	0.0138 (2)	0.0250 (3)	0.00051 (17)	−0.00035 (19)	0.00208 (17)

### Geometric parameters (Å, °)

**Table:**

C1—C2	1.351 (2)	C4—Cl2	1.7151 (18)
C1—Cl1	1.7156 (18)	C4—S1	1.7181 (19)
C1—S1	1.7303 (19)	C5—O1	1.220 (2)
C2—C3	1.437 (2)	C5—C6	1.498 (2)
C2—H2	0.9500	C6—H6A	0.9800
C3—C4	1.376 (2)	C6—H6B	0.9800
C3—C5	1.490 (2)	C6—H6C	0.9800
			
C2—C1—Cl1	127.54 (15)	Cl2—C4—S1	116.58 (10)
C2—C1—S1	112.71 (13)	O1—C5—C3	118.28 (16)
Cl1—C1—S1	119.75 (10)	O1—C5—C6	120.83 (16)
C1—C2—C3	112.84 (16)	C3—C5—C6	120.88 (16)
C1—C2—H2	123.6	C5—C6—H6A	109.5
C3—C2—H2	123.6	C5—C6—H6B	109.5
C4—C3—C2	110.71 (15)	H6A—C6—H6B	109.5
C4—C3—C5	129.40 (16)	C5—C6—H6C	109.5
C2—C3—C5	119.88 (15)	H6A—C6—H6C	109.5
C3—C4—Cl2	130.13 (14)	H6B—C6—H6C	109.5
C3—C4—S1	113.28 (13)	C4—S1—C1	90.45 (8)
			
Cl1—C1—C2—C3	−179.10 (13)	C4—C3—C5—O1	175.49 (17)
S1—C1—C2—C3	0.4 (2)	C2—C3—C5—O1	−3.7 (3)
C1—C2—C3—C4	−0.5 (2)	C4—C3—C5—C6	−4.2 (3)
C1—C2—C3—C5	178.80 (15)	C2—C3—C5—C6	176.57 (15)
C2—C3—C4—Cl2	179.62 (14)	C3—C4—S1—C1	−0.14 (14)
C5—C3—C4—Cl2	0.4 (3)	Cl2—C4—S1—C1	−179.47 (11)
C2—C3—C4—S1	0.40 (19)	C2—C1—S1—C4	−0.18 (14)
C5—C3—C4—S1	−178.85 (14)	Cl1—C1—S1—C4	179.40 (11)
